# Long-term spatial tracking of cells affected by environmental insults

**DOI:** 10.1186/s11689-020-09339-w

**Published:** 2020-12-16

**Authors:** Shahid Mohammad, Stephen J. Page, Toru Sasaki, Nicholas Ayvazian, Pasko Rakic, Yuka Imamura Kawasawa, Kazue Hashimoto-Torii, Masaaki Torii

**Affiliations:** 1 Center for Neuroscience Research, Children’s Research Institute, Children’s National Hospital, Washington, DC USA; 2 Department of Obstetrics and Gynecology, Tokyo Medical University, Tokyo, Japan; 3 Institute of Biomedical Sciences, School of Medicine and Health Sciences, The George Washington University, Washington, DC USA; 4 Department of Neuroscience and Kavli Institute for Neuroscience, Yale University, New Haven, CT USA; 5 Department of Pharmacology, Pennsylvania State University College of Medicine, Hershey, PA USA; 6 Department of Biochemistry and Molecular Biology, Institute for Personalized Medicine, Pennsylvania State University College of Medicine, Hershey, PA USA; 7 Department of Pediatrics, Pharmacology and Physiology, School of Medicine and Health Sciences, The George Washington University, Washington, DC USA

**Keywords:** Environmental stress, Brain development, Heat shock signaling, Reporter, Lineage tracing

## Abstract

**Background:**

Harsh environments surrounding fetuses and children can induce cellular damage in the developing brain, increasing the risk of intellectual disability and other neurodevelopmental disorders such as schizophrenia. However, the mechanisms by which early damage leads to disease manifestation in later life remain largely unknown. Previously, we demonstrated that the activation of heat shock (HS) signaling can be utilized as a unique reporter to label the cells that undergo specific molecular/cellular changes upon exposure to environmental insults throughout the body. Since the activation of HS signaling is an acute and transient event, this approach was not intended for long-term tracing of affected cells after the activation has diminished. In the present study, we generated new reporter transgenic mouse lines as a novel tool to achieve systemic and long-term tracking of affected cells and their progeny.

**Methods:**

The reporter transgenic mouse system was designed so that the activation of HS signaling through HS response element (*HSE*) drives flippase (FLPo)-flippase recognition target (FRT) recombination-mediated permanent expression of the red fluorescent protein (RFP), tdTomato. With a priority on consistent and efficient assessment of the reporter system, we focused on intraperitoneal (i.p.) injection models of high-dose, short prenatal exposure to alcohol (ethanol) and sodium arsenite (ethanol at 4.0 g/kg/day and sodium arsenite at 5.0 mg/kg/day, at embryonic day (E) 12 and 13). Long-term reporter expression was examined in the brain of reporter mice that were prenatally exposed to these insults. Electrophysiological properties were compared between RFP^+^ and RFP^−^ cortical neurons in animals prenatally exposed to arsenite.

**Results:**

We detected RFP^+^ neurons and glia in the brains of postnatal mice that had been prenatally exposed to alcohol or sodium arsenite. In animals prenatally exposed to sodium arsenite, we also detected reduced excitability in RFP^+^ cortical neurons.

**Conclusion:**

The reporter transgenic mice allowed us to trace the cells that once responded to prenatal environmental stress and the progeny derived from these cells long after the exposure in postnatal animals. Tracing of these cells indicates that the impact of prenatal exposure on neural progenitor cells can lead to functional abnormalities in their progeny cells in the postnatal brain. Further studies using more clinically relevant exposure models are warranted to explore this mechanism.

**Supplementary Information:**

The online version contains supplementary material available at 10.1186/s11689-020-09339-w.

## Background

Exposure to environmental insults such as chemicals or infectious agents during development alters the molecular properties of afflicted cells [1], affecting various critical cellular processes including those in brain development [2–6], and increasing the risks of neuropsychiatric disorders [7]. However, the mechanisms by which environmental impact during the early developmental period leads to variable disease manifestation in later life remain largely unknown.

Activation of HS signaling, which is not limited by heat stress alone but by various types of environmental stress, is one of the intrinsic cytoprotective mechanisms known to protect the developing brain from deleterious outcomes [8–10]. We previously generated reporter transgenic mice, in which heat shock factor 1 (Hsf1)-mediated acute activation of HS signaling is visualized by the expression of RFP reporter under the control of the HS response element (*HSE*) that contains the mouse *Hsp70* promoter [11, 12]. Using these mice, we have shown that the acute activation of HS signaling in the embryonic brain upon exposure to environmental insults, such as alcohol and sodium arsenite, is heterogeneous among neural progenitor cells and occurs in a stochastic manner [11, 12]. We have also demonstrated that cells labeled by reporter expression exhibit deficits in cell cycling and migration during embryonic cortical development [11, 12], indicating that the cells which underwent HS signaling activation and survived the exposure may contribute to brain malfunction in later life. It has also been shown that HS signaling can mark epigenetic transcriptional memory, and thereby causes long-term changes in gene expression [13]. These lines of evidence prompt the need for a novel tool that can track these affected cells and their progeny, and address the mechanism underlying long-term consequences of prior impacts. The reporter transgenic mice that we previously generated [11, 12], however, were not specifically designed for such a tracing purpose, and only retain reporter expression for a few days after stress exposure.

In the present study, we report the generation of novel reporter transgenic mice that harbor a combination of the *HSE*-driven *FLPo* and *FRT-RFP* transgenes for long-term lineage tracing of cells in which HS signaling was activated. This novel animal model allowed us to explore the impact of prenatal alcohol or arsenic exposure on neuronal functions in the cerebral cortex of postnatal animals.

## Methods

### Animals and drug administration

All mice were maintained on a light–dark cycle (lights on 06:00–18:00 h) at a constant temperature (22 ± 1 °C). Pregnant mice received i.p. injections of 25% ethanol in PBS at 4.0 g/kg/day or sodium arsenite in PBS at 5.0 mg/kg/day at E12 and E13. PBS alone was injected as the control for immunohistochemistry experiments. Untreated animals were used as controls for electrophysiology experiments. Weights of carcasses, brains, and lungs were measured at necropsy. All protocols were approved by the Institutional Animal Care and Use Committee (IACUC) of the Children’s National Hospital. All methods were performed in accordance with relevant guidelines and regulations.

### Generation of the HSE-FLPo mouse

Using Vista Genome Browser (pipeline.lbl.gov/cgi-bin/gateway2), the oligonucleotide including HSE [12] and codon-optimized flippase (FLPo) [14] sequence was synthesized and cloned into pUC57 (Genscript). Kpn1 and Sma1 recognition sites were added at the ends of the following 2252-bp sequence for synthesis.

HSE-FLPo:CAGCTTCACCCACAGGGACCCCGAAGTTGCGTCGCCTCCGCAACAGTGTCAATAGCAGCACCAGCACTTCCCCACACCCTCCCCCTCAGGAATCCGTACTCTCTAGCGAACCCCAGAAACCTCTGGAGAGTTCTGGACAAGGGCGGAACCCACAACTCCGATTACTCAAGGGAGGCGGGGAAGCTCCACCAGACGCGAAACTGCTGGAAGATTCCTGGCCCCAAGGCCTCCTCCGGCTCGCTGATTGGCCCAGCGGAGAGTGGGCGGGGCCGGTGAAGACTCCTTAAAGGCGCAGGGCGGCGAGCAGGGCACCAGACGCTGACAGCTACTCAGAATCAAATCTGGTTCCATCCAGAGACAAGCGAAGACAAGAGAAGCAGAGCGAGCGGCGCGTTCCCGATCCTCGGCCAGGACCAGCCTTCCCCAGAGCATCCACGCCGCGGAGCGCAACCTTCCCAGGAGCATCCCTGCCGCGGAGCGCAACTTTCCCCGGAGCATCCACGCCGCGGAGCGCAGCCTTCCAGAAGCAGAGCGCGGCGCCATGGCCAAGAACACGGCGATCGGCATCGACCTGGGCACCACCTACTCGTGCGTGGGCGTGTTCCAGCACGGCAAGGTGGAGATCATCGCCAACGACCAGGGCAACCGCCGGTGGCGGCCGCGATCAAGCTTCTGCAATCGCCGCCACCATGGCTCCTAAGAAGAAGAGGAAGGTGATGAGCCAGTTCGACATCCTGTGCAAGACCCCCCCCAAGGTGCTGGTGCGGCAGTTCGTGGAGAGATTCGAGAGGCCCAGCGGCGAGAAGATCGCCAGCTGTGCCGCCGAGCTGACCTACCTGTGCTGGATGATCACCCACAACGGCACCGCCATCAAGAGGGCCACCTTCATGAGCTACAACACCATCATCAGCAACAGCCTGAGCTTCGACATCGTGAACAAGAGCCTGCAGTTCAAGTACAAGACCCAGAAGGCCACCATCCTGGAGGCCAGCCTGAAGAAGCTGATCCCCGCCTGGGAGTTCACCATCATCCCTTACAACGGCCAGAAGCACCAGAGCGACATCACCGACATCGTGTCCAGCCTGCAGCTGCAGTTCGAGAGCAGCGAGGAGGCCGACAAGGGCAACAGCCACAGCAAGAAGATGCTGAAGGCCCTGCTGTCCGAGGGCGAGAGCATCTGGGAGATCACCGAGAAGATCCTGAACAGCTTCGAGTACACCAGCAGGTTCACCAAGACCAAGACCCTGTACCAGTTCCTGTTCCTGGCCACATTCATCAACTGCGGCAGGTTCAGCGACATCAAGAACGTGGACCCCAAGAGCTTCAAGCTGGTGCAGAACAAGTACCTGGGCGTGATCATTCAGTGCCTGGTGACCGAGACCAAGACAAGCGTGTCCAGGCACATCTACTTTTTCAGCGCCAGAGGCAGGATCGACCCCCTGGTGTACCTGGACGAGTTCCTGAGGAACAGCGAGCCCGTGCTGAAGAGAGTGAACAGGACCGGCAACAGCAGCAGCAACAAGCAGGAGTACCAGCTGCTGAAGGACAACCTGGTGCGCAGCTACAACAAGGCCCTGAAGAAGAACGCCCCCTACCCCATCTTCGCTATCAAGAACGGCCCTAAGAGCCACATCGGCAGGCACCTGATGACCAGCTTTCTGAGCATGAAGGGCCTGACCGAGCTGACAAACGTGGTGGGCAACTGGAGCGACAAGAGGGCCTCCGCCGTGGCCAGGACCACCTACACCCACCAGATCACCGCCATCCCCGACCACTACTTCGCCCTGGTGTCCAGGTACTACGCCTACGACCCCATCAGCAAGGAGATGATCGCCCTGAAGGACGAGACCAACCCCATCGAGGAGTGGCAGCACATCGAGCAGCTGAAGGGCAGCGCCGAGGGCAGCATCAGATACCCCGCCTGGAACGGCATCATCAGCCAGGAGGTGCTGGACTACCTGAGCAGCTACATCAACAGGCGGATCTGATGAGATATCCTAGAGCTCGCTGATCAGCCTCGACTGTGCCTTCTAGTTGCCAGCCATCTGTTGTTTGCCCCTCCCCCGTGCCTTCCTTGACCCTGGAAGGTGCCACTCCCACTGTCCTTTCCTAATAAAATGAGGAAATTGCATCGCATTGTCTGAGTAGGTGTCATTCTATTCTGGGGGGTGGGGTGGGGCAGGACAGCAAGGGGGAGGATTGGGAAGACAATAGCAGGCATGCTGGGGATGCGGTGGGCTCTATGGCTTCTG.

**Fig. 1:**
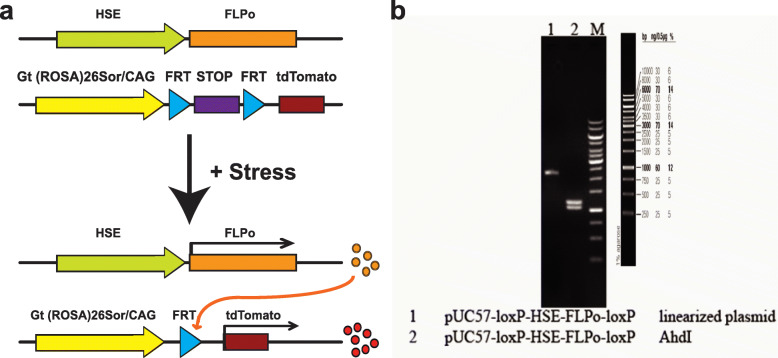
The reporter construct for long-term detection of cell lineages that experienced HSF1 activation. **a** The design of RFP reporter system, in which the *HSE* and FLPo-FRT recombination system are combined for long-term labeling. Upon exposure to stress, *HSE* drives the expression of FLPo, which then induces RFP expression via FRT recombination. **b** The *HSE-FLPo* fragment (2301 bp) was verified by AhdI digestion that generated the expected 1216 and 1085 bp products

To generate *HSE-FLPo* transgenic mice, the *HSE-FLPo* fragment (2301 bp) was excised using KpnI, SmaI, and ScaI and purified according to the standard protocol for microinjection. Prior to the injection, the 2301-bp band was further verified with the expected 1216- and 1085-bp products of AhdI digestion (Fig. 1b). Microinjection into the C57BL/6 J X C57BL/6 J strain was performed by Cyagen. Eleven out of 63 founder lines were screened by PCR genotyping for FLPo. The tails of the 11 pups were re-cut and the insertion of transgene was confirmed by PCR (Fig. S1) using the following primers: transgene primers F1: AGATCATCGCCAACGACCAG and R1: TAAGGGATGATGGTGAACTCCCAG (product size: 393 bp); internal control primers F1: CTATCAGGGATACTCCTCTTTGCC and R1: GATACAGGAATGACAAGCTCATGGT (product size: 507 bp); transgene primers F2: AGGCACCTGATGACCAGCTTTC and R2: ACAACAGATGGCTGGCAACTAG (product size: 415 bp); internal control primers F2: GCAGAAGAGGACAGATACATTCAT and R2: CCTACTGAAGAATCTATCCCACAG (product size: 689 bp). The internal control PCR targets the endogenous mouse Rgs7 (G protein signaling 7) locus. PCR was carried out in 25 μL volume for 38 cycles with the annealing temperature at 60 °C under standard conditions, with all four primers listed above added to each reaction. Taq DNA polymerase used was TaKaRa R004A. Three controls used in PCR genotyping were (a) water control: no DNA template added, (b) positive control: 400 ng of mouse genomic DNA spiked with an amount of transgene injection DNA that was equivalent to 5 copies of transgene per diploid mouse genome, and (c) wildtype control: 400 ng of mouse genomic DNA.

The 11 founder lines (A1-A11) were then separately crossed with *RC::FLTG* mice [*B6.Cg-Gt(ROSA)26Sortm1.3(CAG-tdTomato,-EGFP)Pjen/J*, Stock No: 026932, The Jackson Laboratory], in which FLPo-FRT recombination results in high expression of tdTomato, and F1 generations were exposed to ethanol or sodium arsenite at E12 and E13 to confirm the reporter expression. One of the three founder lines that showed the most consistent expression of reporter was further examined in this study. The routine genotyping of double transgenic animals was performed by Transnetyx.

### Whole-genome sequencing of HSE–RFP transgenic mouse

Shallow whole-genome sequencing of the genomic DNA extracted from transgenic mice was performed using an Illumina NovaSeq 6000 at ~ 151-bp paired-end aiming at ∼ 3 × whole-genome coverage [15]. To identify candidate integration sites, paired-end reads were aligned to the reference mouse genome using Bowtie2 [16] in global (reads align end to end) and local (reads are soft-clipped) modes allowing 0 or 1 mismatches for potential SNPs or PCR errors [17, 18], and discordant read pairs were reported. As an alternative approach, we also ran Burrows–Wheeler Aligner (BWA) using the BWA-MEM (maximum exact matches), which performs local alignment and supports split (chimeric) read alignments [18, 19]. To calculate the depth of sequencing coverage, we used GATK DepthofCoverage tool with the mouse reference genome (GRCm38).

### Validation of transgene insertion loci by polymerase chain reaction

To validate the transgene insertion in chromosomes 18 and 19, polymerase chain reaction was performed using the following pairs of primers designed within the FLPo sequence or intronic regions flanking the insertion site: FLPoF: TGATGAGCCAGTTCGACATC and chr18R: CCCTGCCAGAGACAGACAAC (for chromosome 18), chr19F: TGAGCCTTTCTGGCTTCAGG and chr19R: TGAGCCTTTCTGGCTTCAGG (for chromosome 19). Genomic DNA was extracted from the brain or tail tissue using MinElute PCR Purification Kit (Qiagen). PCR was performed using Takara PCR master mixes (chromosome 18) or Promega GoTaq (chromosome 19) with 35 cycles at annealing temperature of 55 °C. PCR products were detected using the conventional agarose gel electrophoresis method or BioAnalyzer (Agilent Technologies).

### Immunohistochemistry

Mice were perfused with 4% paraformaldehyde (PFA) following a standard protocol. The brains were collected and post-fixed in 4% PFA at 4 °C overnight, followed by incubation in 10% and 30% sucrose in PBS at 4 °C for 24 h. Coronal sections (60 μm thickness) were made using a cryostat (CM3050S; Leica). Free-floating sections were treated by hydrogen peroxide in methanol (1:4) solution at − 20 °C for 20 min to inactivate endogenous peroxidase activity. After washing with PBS-T (PBS containing 0.2% Tween 20) three times, sections were incubated with blocking buffer [2% bovine serum albumin (BSA) in PBS-T] for 30 min at room temperature. Sections were then incubated with a rabbit anti-RFP antibody diluted in the blocking buffer (1:500) (Abcam) overnight at 4 °C. Incubation with a horseradish peroxidase (HRP)-conjugated anti-rabbit IgG (1:500) (Jackson ImmunoResearch) was for 3 h at room temperature. TSA kit (PerkinElmer) was used for signal amplification. DAPI (4′,6-diamidino-2-phenylindole, dihydrochloride) (1:10,000) (Sigma–Aldrich) was used for nuclear counterstaining. Labeled sections were imaged using an Olympus confocal microscope equipped with a digital camera.

### Coronal slice preparation for electrophysiology

Reporter transgenic mice (P15–21) were used for electrophysiology experiments. No significant differences were observed on the basis of sex, and therefore data collected from males and females were combined for analysis. Mice were anesthetized by inhalation of isoflurane (2–4%) and decapitated. The forebrain was removed and placed for 1 min in cold (0–4 °C) artificial cerebrospinal fluid (aCSF) composed of the following (in mM): NaCl 125, KCl 3, KH_2_PO_4_ 1.2, MgSO_4_ 1.2, NaHCO_3_ 25, dextrose 10, CaCl_2_ 2, and bubbled with 95% O_2_/5% CO_2_ (pH 7.4, 306–310 mOsM adjusted with dextrose). Using a vibrating microtome (Leica VT-1000S), 300-μm-thick coronal forebrain slices were cut. Slices were incubated at room temperature in a submersion chamber containing an identical oxygenated aCSF for at least 45 mins before use.

### Electrophysiology

Cortical neurons expressing RFP were visually selected for electrophysiological patch-clamp recordings using a fluorescence microscope (Olympus BX51WI). Patch electrodes were guided to neurons using differential interference contrast (DIC) optics illuminated with infrared light. Voltage–clamp and current–clamp recordings were made with a MultiClamp 700B amplifier, Digidata 1440A digitizer, and pClamp10.7 software (all from Molecular Devices). Data were filtered at 10 kHz and stored on a personal computer for offline analysis. For recordings, patch pipettes were pulled using a horizontal pipette puller (P-2000, Sutter Instruments). The pipette resistance was 6.0–9.0 MΩ when filled with internal solution (in mM: NaCl 10, K-gluconate 130, EGTA 11, CaCl_2_ 1, MgCl_2_ 2, HEPES 10, Na-ATP 2, Na-GTP 0.2, pH 7.3, 297–300 mOsM). After formation of a stable gigaohm seal (> 1 GΩ), the whole-cell configuration was established. Only neurons with holding currents not exceeding ± 200 pA at *V*_Hold_ = − 60 mV for the 10-min control period were studied further. Access resistance (Ra) was monitored throughout recordings, and neurons were not included in additional analysis if it exceeded 10 MΩ or drifted > 20%. Whole-cell recordings were made in extracellular solution that was identical to oxygenated aCSF used for brain tissue preparation. aCSF was heated to 32 ± 0.5 °C using a pre-heater, temperature sensor (positioned on the wall of the recording chamber next to the slice), and temperature controller (Warner Instruments).

### Data analysis

All values are reported as mean ± SEM unless otherwise indicated. In box plots, the line within the box indicates the median, and the upper and lower edges of the box represent the 25th and 75th percentiles, respectively. The upper and lower whisker boundaries indicate the 10th and 90th percentiles, respectively. All quantitative data were statistically analyzed using chi-square test or one-way analysis of variance (ANOVA) (with repeated measures where appropriate), using GraphPad Prism version 7 (GraphPad Software) or Origin 9 software (OriginLab). *P* < 0.05 values were considered statistically significant.

## Results

### Generation of reporter transgenic mice for long-term labeling of cell lineages in which heat shock signaling is activated

To generate reporter transgenic mice for in vivo long-term tracking of cell lineages that responded to environmental insults via activation of HS signaling, we designed a reporter system in which the activation of HS signaling drives the FLPo-FRT recombination-mediated expression of RFP (Fig. 1). A similar reporter system has been validated in vivo using in utero electroporation-mediated gene transfer method in our previous studies [12, 20]. For the 11 founder lines we obtained, we confirmed the insertion of the *HSE-FLPo* transgene by PCR (Fig. S1). After crossing these *HSE-FLPo* transgenic mice with *RC::FLTG* mice, in which RFP (tdTomato) expression is triggered by FLPo-FRT recombination, we examined RFP expression in the brains of adolescent mice (at P20) born from dams that had been administered alcohol (4.0 g/kg/day) during embryonic days (E) 12 and 13. Of the three *HSE-FLPo* founder lines (lines A4, A6, and A8), in which we observed strong expression of RFP in the brain, one line (A4) was further analyzed. Shallow whole-genome sequencing was performed to define the loci of transgenes in the F2 generation. 95% of the genome was covered at least 1×, and the mean mapped read depth was 3.54× with an interquartile range of 3, suggesting enough coverage to detect chromosomal structural variations [21]. The sequencing confirmed that the genomic loci of inserted transgene were located in intronic regions in the direction opposite to DNA transcription (Figs. S2, S3), suggesting a low possibility of disturbance in intrinsic gene expression in the reporter mice. After several generations of breeding (after the F5 generation), polymerase chain reaction (PCR) was performed to further validate the transgene insertion. The results indicated that these mice inherited and maintained the transgene only in chromosome 18 in a stable manner (Figs. S2, S3). All the following studies were performed using this mouse line (A4) that maintains the transgene only in chromosome 18.

### Tracking of the cell lineages affected by prenatal exposure to environmental insults in the postnatal brain

To test whether the new reporter mice allow long-term tracing of cell lineages affected by environmental insults, we used models of prenatal exposure to alcohol and arsenic as two representative environmental insults and examined the consequence postnatally. Prenatal alcohol (ethanol) exposure due to maternal consumption of alcohol during pregnancy causes a broad range of adverse developmental effects, including physical, behavioral, and learning problems that are collectively referred to as fetal alcohol spectrum disorders (FASD) [22, 23]. Arsenite is an inorganic form of arsenic, a metalloid naturally found in soil, air, and water in some zones of the world. Cumulative evidence from epidemiological and animal studies indicates that arsenic exposure in utero or in early childhood causes developmental neurotoxicity, affecting intellectual functions throughout life [24–29].

**Fig. 2:**
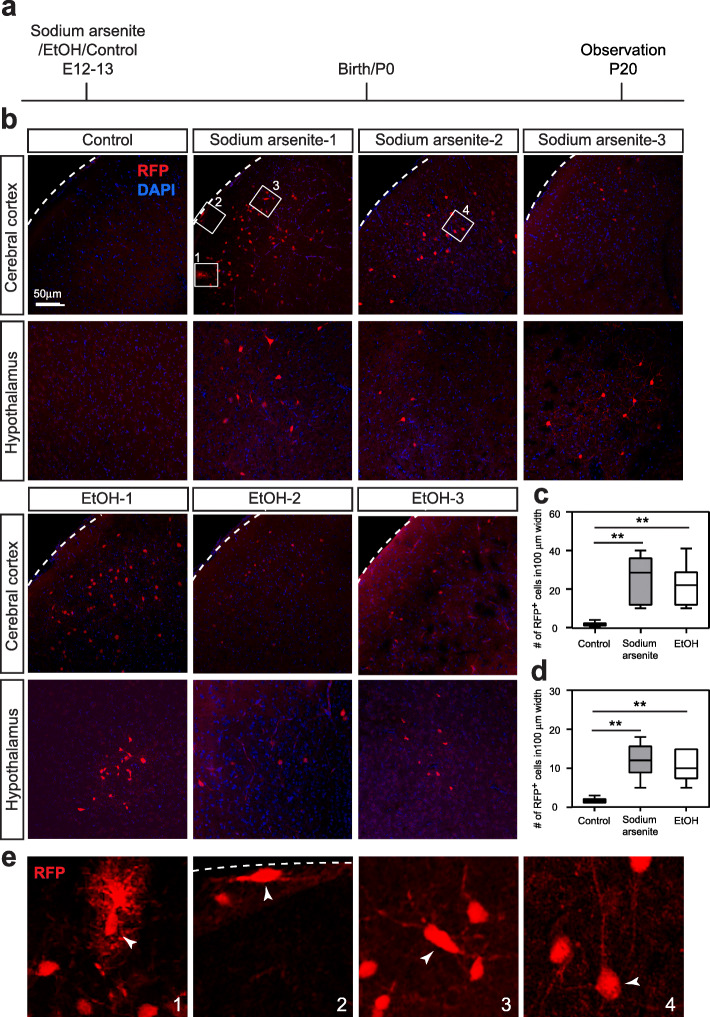
RFP reporter expression in the postnatal brain of reporter transgenic mice prenatally exposed to sodium arsenite or ethanol. **a** Experimental timeline. **b** Immunohistochemistry for RFP in the cerebral cortex and hypothalamus of the reporter transgenic mice (line A4) that were prenatally exposed to PBS (control), sodium arsenite, or ethanol (EtOH) as indicated. Results from 3 different animals each are shown for sodium arsenite- and ethanol-exposed groups. Broken lines indicate the pial surface. Sections were counterstained with DAPI. Observations were made at P20. **c** The average density of RFP^+^ cells (in coronal sections) throughout the cerebral cortex; F(2, 15) = 10.66, *P* = 0.001 by one-way ANOVA, **P < 0.01 by Tukey test. **d** The average density of RFP^+^ cells (in coronal sections) throughout the hypothalamus; F(2, 15) = 15.69, *P* = 0.0002 by one-way ANOVA, **P < 0.01 by Tukey test. **e** Higher magnification views of the boxed areas 1–4 in (**b**). Pial surface is oriented to the top. Reporter expression was observed in various cell types in the cerebral cortex, including glial (1), presumptive Cajal–Retzius cell (2), interneuron (3), and pyramidal neuron (4) (arrowheads). The broken line indicates the pial surface

**Fig. 3:**
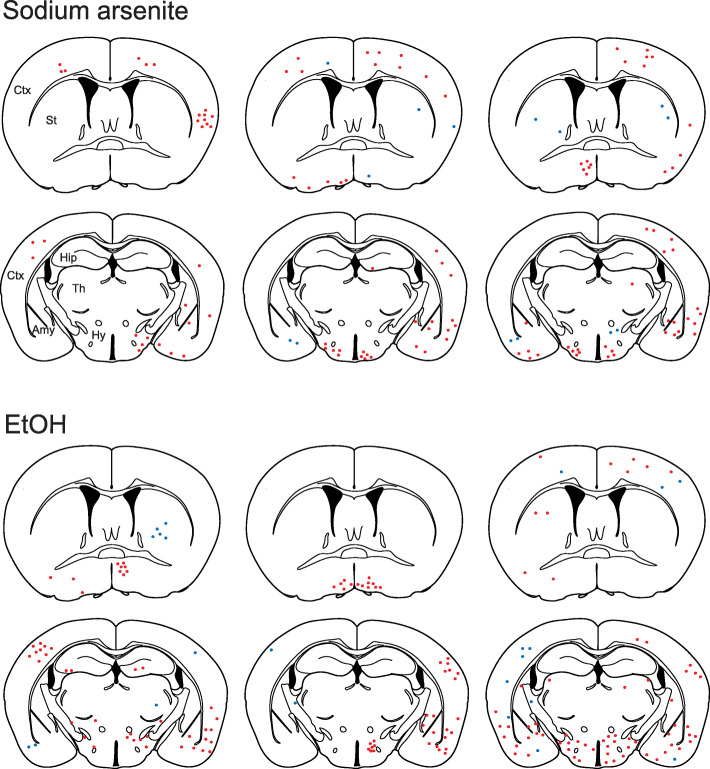
Schematic representation of the distribution of RFP-positive cells in the anterior and posterior parts of the forebrain in sodium arsenite- and ethanol-exposed mice. Results from three P20 mice each are shown. Red dots and blue dots indicate, neurons and glial cells, respectively. Each dot represents 2–3 cells. Ctx: cerebral cortex; Hip: hippocampus; St: striatum; Th: thalamus; Hy: hypothalamus; Amy: amygdala

For efficient assessment of the reporter system, we focused on high-dose, short exposure models via i.p. injections; pregnant transgenic mice were administered ethanol (4.0 g/kg/day) or sodium arsenite (5.0 mg/kg/day) (or PBS as control) at E12 and E13. With these conditions, no obvious toxicity was observed in dams and pups. Body weight and major organ weights were not affected in the pups at P20 (Fig. S4). In both ethanol- and arsenite-exposed pups, but not in PBS-exposed pups, we observed RFP expression in neurons and glial cells scattered across various brain regions, including the cerebral cortex, hippocampus, striatum, thalamus, and hypothalamus (Figs. 2 and 3). The expression patterns were different between individual animals (Figs. 2 and 3), consistent with the stochastic pattern of HS signaling activation by exposure to environmental insults in neural progenitor cells in the embryonic brain [10, 12, 30].

### Abnormal electrophysiological properties of reporter-positive neurons in the brain of postnatal animals that were prenatally exposed to environmental insults

**Fig. 4:**
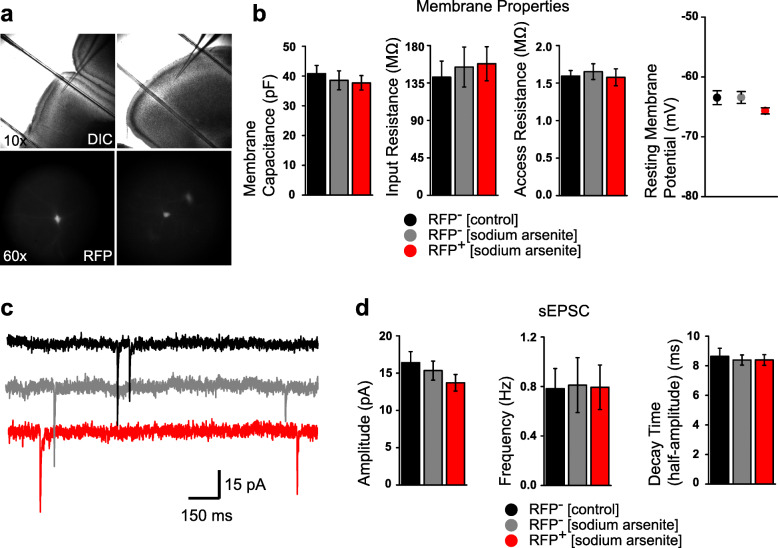
Functional characteristics of RFP-positive cortical pyramidal neurons. **a** Representative DIC images of patch-clamp experiments conducted on upper layer cortical pyramidal neurons (top), and fluorescent images of RFP expressing neurons used for recording (bottom). **b** No significant differences were observed in neuronal membrane properties including: capacitance [F(2, 52) = 0.16, *P* = 0.85 by one-way ANOVA], input resistance [F(2, 52) = 0.077, *P* = 0.93], access (series) resistance [F(2, 52) = 0.09, *P* = 0.91], and resting potential [F(2, 24) = 0.63, *P* = 0.54] between RFP^+^, RFP^−^, and RFP^−^ control neurons. **c** Example voltage-clamp traces depicting spontaneous excitatory post-synaptic currents (sEPSC) recorded in cortical neurons from each group in reporter transgenic mice. **d** No significant differences were observed in sEPSC characteristics including amplitude [F(2, 31) = 0.65, *P* = 0.53 by one-way ANOVA], frequency [F(2, 31) = 0.004, *P* = 0.99], and decay time (half-amplitude) [F(2, 31) = 0.08, *P* = 0.92] between groups

To test whether the impact of prenatal exposure, marked by the activation of HS signaling in neural progenitors, alters electrophysiological properties in their progeny after birth, embryos of reporter transgenic mice were exposed to sodium arsenite during E12 and E13 by i.p. injecting pregnant dams with sodium arsenite (5.0 mg/kg/day). Offspring were sacrificed for acute brain slice whole-cell recording from cortical pyramidal neurons in the primary motor cortex (layers II and III) at P15–21 (Fig. 4a). Neurons negative for RFP expression, from sodium arsenite-treated or untreated mice were recorded as controls. Voltage-clamp recordings revealed no overall differences in neuronal membrane properties between RFP^+^ and RFP^−^ pyramidal neurons (Fig. 4b). Additionally, recordings performed at − 60 mV to evaluate spontaneous excitatory post-synaptic currents (sEPSC) showed no differences between any of the three groups of neurons (Fig. 4 c and d), indicating that fast-excitatory synaptic transmission was not altered by prenatal exposure to sodium arsenite.

**Fig. 5:**
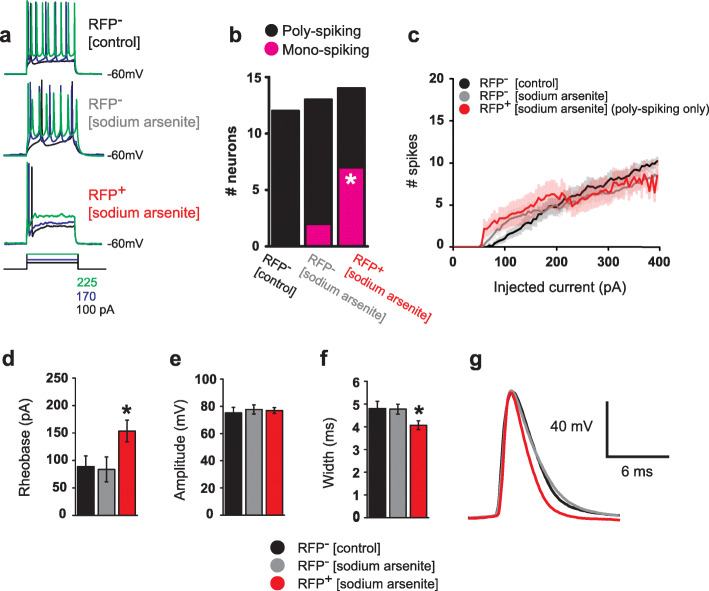
RFP-positive cortical pyramidal neurons exhibit reduced excitability. **a** Representative current–clamp traces of action potentials (AP) evoked by current injection at rheobase (black) and above threshold (blue and green). While AP frequency generally increased with step amplitude in RFP^−^ neurons from either control- or arsenite-treatment groups, RFP^+^ neurons tended to elicit single spikes regardless of stimulus intensity (bottom trace). **b** Quantification of mono-spiking neurons recorded in each condition. A larger proportion of RFP‑expressing neurons were classified as mono-spiking when compared to both RFP-negative groups [*X*^2^ (1, 40) = 7.38, *P* < 0.05 by chi-square test]. **c** Stimulus-frequency curves show that poly-spiking RFP^+^ neurons (red) follow a profile similar to RFP^−^ neurons from both arsenite and untreated groups (black and gray) [F(1, 99) = 0.34, *P* = 0.71 by one-way repeated measures ANOVA]. **d** The average amount of depolarizing current required to elicit an action potential from rest (rheobase) was increased in RFP^+^ neurons [F(2, 30) = 3.33, *P* < 0.05 by one-way ANOVA, *P < 0.05 by Tukey test]. **e** No differences were observed in the peak amplitude of 1st APs between groups [F(2, 29) = 0.17, *P* = 0.86 one-way ANOVA]. **f** RFP+ neurons elicited narrower 1st APs when compared to RFP^−^ neurons from both arsenite and untreated groups [F(2, 31) = 6.13, *P* < 0.005 by one-way ANOVA, *P < 0.05 by Tukey test]. **g** Representative spike traces from individual RFP^+^, RFP^−^, and control neurons demonstrating that 1st AP width is reduced in RFP^+^ neurons, without a change in peak amplitude

To investigate whether action potential (AP) firing is disrupted by sodium arsenite exposure during pregnancy, we performed current–clamp recordings in RFP^+^ and RFP^−^ neurons from reporter transgenic mice that were exposed to sodium arsenite prenatally, as well as in RFP^−^ neurons from untreated reporter transgenic mice. Recorded neurons were maintained at a holding potential of − 60 mV, and a series of current steps (− 100 pA to 395 pA, 500 ms, 5 pA steps) were applied to study AP firing across a range of stimulation intensities. It was found that, while RFP^−^ neurons, from both sodium arsenite and untreated groups, tended to follow a typical stimulation–frequency curve, a large portion of RFP^+^ neurons exhibited mono-spiking, in which a single AP was evoked by a current step at threshold, and for all stimulus intensities above firing threshold (Fig. 5a). Out of all RFP^+^ neurons tested, 50% (7/14) were classified as mono-spiking neurons, whereas only 15% (2/13) of RFP^−^ neurons from sodium arsenite treated mice, and none (0/12) of the RFP^−^ neurons tested in untreated mice produced single-spikes (Fig. 5b). Among the RFP^+^ neurons that did engage in typical spiking behavior (7/14 poly-spiking), no differences were found in average stimulation–frequency curve when compared to RFP^−^ neurons from either sodium arsenite or untreated groups (Fig. 5c). Finally, it was found that RFP^+^ neurons required a greater amount of current injection to reach the AP firing threshold (Fig. 5d) and produced slightly narrower 1st APs (Fig. 5 f and g) without a change in 1st AP amplitude (Fig. 5e). Together, our results indicate that excitability is generally reduced in RFP+ neurons in the arsenite-exposed cortex, as they are significantly more likely to produce single, narrow spikes at an increased current threshold.

## Discussion

In the present study, we report that our novel transgenic mouse line enables long-term lineage tracing of cells that had once activated HS signaling to respond to environmental insults, using two different prenatal exposure models (alcohol and arsenic exposure).

### Tracing the lineage of cells prenatally affected by environmental insults

Prenatal exposure to alcohol and arsenic has been shown to cause developmental neurotoxicity, affecting intellectual, emotional, and social functions permanently throughout later life [22, 24–29]. In the reporter transgenic mice, we observed RFP reporter expression, which reflects prior activation of HS signaling, in both neurons and glial cells broadly in the postnatal brain. The affected brain regions include the cerebral cortex and hypothalamus, in which the impact of early exposure to alcohol and arsenic has been suspected to contribute to cognitive and behavioral deficits [31–35].

Our previous studies have shown that HSE drives reporter expression not only in the brain but also in many organs and tissues throughout the body of the embryo, including the eyes, heart, lung, and limb bud, in response to prenatal exposure to environmental insults [12]. Detection of cellular damage is also not limited to that of prenatal insults, but includes postnatal insults such as spinal cord or sciatic nerve injury [36]. Therefore, it is expected that these transgenic mice enable long-term tracing of neural as well as non-neural cell lineages affected by various types of chemical, infectious, or physical environmental factors during both prenatal and postnatal periods. The reporter transgenic mice were generated by crossing the *HSE-FLPo* mice with the *RC::FLTG* mice, which can also express enhanced green fluorescent protein (EGFP) in a Cre recombinase-dependent manner. This will help to investigate RFP reporter-positive and -negative cells within EGFP-labeled specific cell populations.

### Functional impact of prenatal exposure to environmental insults on cortical neurons

In our study of cortical neurons, we tested for physiological alterations that were specific to neuronal lineages that underwent HS activation at their progenitor stage as a result of sodium arsenite exposure. We found that intrinsic cell membrane properties were identical between RFP^+^ and RFP^−^ pyramidal neurons in layers II and III, indicating that HS activation, and the associated impact of arsenite exposure during pregnancy, are unlikely to bestow obvious long-term changes to cortical cell membranes in offspring. Likewise, the membrane properties of RFP^+^ neurons were found to be no different from RFP^−^ neurons recorded in untreated mice, suggesting no obvious effect of prenatal stressor (sodium arsenite) on membrane properties in mature cortical neurons. Additionally, spontaneous excitatory inputs onto RFP^+^ cells appeared to be unchanged compared to RFP^−^ cells from both arsenite-treated and untreated mice, indicating no effect of prenatal HS activation or sodium arsenite exposure on the rate of vesicle release at glutamatergic synapses, or on post-synaptic signaling mechanisms such as glutamate receptor expression/function.

Despite the numerous consistencies we observed between RFP^+^ and RFP^−^ cortical pyramidal neurons, current–clamp recordings revealed a distinct phenotype of reduced excitability in RFP^+^ cells. When subjected to depolarizing current injection, half of RFP^+^ neurons elicited only a single action potential, rather than a train of spikes, at stimulations well above the firing threshold—a phenomenon that was not observed in control neurons. Interestingly, a similar low-excitability phenotype was reported in lower layer cortical pyramidal neurons as a result of early ethanol exposure, where single-spiking was also prominent [37]. This restricted form of action potential generation, associated with increased rheobase current, narrowed spike width, and no change in resting potential, input resistance, or AP amplitude, can be attributed to low threshold, slowly inactivating, potassium (K^+^) currents as described previously [38–42]. In the cortex, such hyperpolarizing K^+^ currents are typically mediated by Kv1.1 subunit-containing voltage-gated K^+^ channels, which activate near the AP threshold (~ − 45 mV) and serve to prevent repetitive firing during a prolonged stimulus [43]. In the context of our data, it is possible that Kv1.1 channels have become upregulated following environmental stress, specifically in RFP^+^ neurons, and enforce single-spike responses to membrane depolarization. Future studies might test this hypothesis by assessing the ability of Kv1-selective channel blockers to reverse the low output phenotype observed in RFP^+^ neurons.

It is well known that arsenic negatively impacts brain development, as rodent studies have reported deficits in neuromotor development [44, 45] and cognitive flexibility [24] as consequences of prenatal exposure. Our finding, that excitability is reduced among pyramidal neurons located in layers II and III of the primary motor cortex, provides a possible explanation for arsenite-induced impairments, as it represents a striking loss of output from a cell population that is critical for coordinating sensorimotor responses [46]. Importantly, the observed single-spiking phenotype was specific to neurons that expressed the RFP reporter and had therefore undergone HS activation earlier in their lineage. Thus, our results point to HS signaling-activated neural populations as promising therapeutic targets for the treatment of prenatal arsenic exposure. Other important questions would be whether abnormal characteristics are found commonly in reporter-positive cells in brains exposed to different types of environmental stress, and what behavioral consequences are brought by observed cellular changes. Given our results were obtained under extreme exposure conditions (i.p. injections at high doses for a short period), studies using different, more clinically relevant conditions (e.g., chronic drinking model) would also be necessary.

### Mechanisms for the long-term effects of prenatal exposure

As discussed above, it is likely that the long-term impact of prenatal exposure to environmental insults is mediated by altered regulation of key molecules, such as voltage-gated ion channels, in the mature brain. Defining the pathological molecular profiles in reporter-positive cells would be of particular interest. One possible mechanism for long-term changes in gene regulation is that earlier impacts of exposure and HS activation in neural progenitor cells cause epigenetic modifications that are inherited by mature neurons originated from these progenitor cells. In fact, modification of epigenetic signatures by prenatal exposure to alcohol [47–50] and arsenic [51–53] has been shown and suggested to have permanent consequences on brain development. HS signaling has also been shown to affect epigenetic regulations, causing long-term changes in gene expression [13, 54]. Elucidating the epigenetic mechanisms that may underlie long-term adverse outcomes of prenatal exposure to environmental insults will facilitate our research toward the discovery of novel therapeutic approaches for intellectual disability and other neuropsychiatric disorders. It would be also important to expand similar approaches to other stress response pathways, such as the ER stress response pathway [55, 56], to uncover the whole picture of cellular damage established in utero and propagated throughout life.

## Conclusions

Here, we report the generation and use of new reporter transgenic mice that enable long-term tracing of cell lineages that once have responded to environmental insults, via activation of HS signaling. This new transgenic mouse line will serve as a versatile tool to fill the gap in our knowledge on the mechanisms underlying the long-term impacts of environmental insults that often manifest as intellectual disability or other neurodevelopmental disorders long after the actual exposure.

## Supplementary Information

**Additional file 1: Supplementary Figure 1.** Confirmation of transgene insertion in the founder lines. PCR was performed with two different primer sets using the tail tissue of founder lines (numbers 1–11 for lines A1-A11, respectively) for detection of the transgene (393 bp in upper panel, 415 bp in lower panel). The *Rgs7* locus was used as the internal control (507 bp in upper panel, 689 bp in lower panel).

**Additional file 2: Supplementary Figure 2.** Genomic locus of the transgene integration in chromosome 18 in the founder line A4. (a) Whole genome sequencing with 3X coverage defined an intronic region of *Tcerg1* gene locus in chromosome 18 as the region of the transgene insertion in the F2 generation. The direction of the transgene in intronic regions is opposite to the transcriptional direction of the endogenous gene. Each one of the paired reads includes a partial sequence of *FLPo*. (b) PCR using the primer pair of FLPoF/chr18R after several generations of breeding (after the F5 generation) resulted in a ~ 1500 bp product (arrow) from the sample of founder line A4, confirming the transgene insertion in the identified locus. No PCR product was amplified from the sample of wild-type (WT) mice. Lane 1–4: DNA ladder, A4 genomic DNA input, A4 PCR product, and WT PCR product, respectively.

**Additional file 3: Supplementary Figure 3.** Genomic locus of the transgene integration in chromosome 19 in the founder line A4. (a) Whole genome sequencing identified transgene insertion also in the 29 intronic region of *Ablim1* gene in chromosome 19 in the F2 generation of A4. The direction of the transgene is opposite to the transcriptional direction of the endogenous gene. (b) PCR was performed to validate the insertion on the transgene in the founder line A4 using the primer pair of chr19F/chr19R after several generations of breeding (after the F5 generation). The size of PCR product detected by the BioAnalyzer was ~ 600 bp (arrow), which is expected to be amplified from the intrinsic genomic locus, as shown by the same size of product also amplified from the WT sample. Consistently, the product of > 2000 bp, which is expected to be amplified if the transgene is inserted, was not detected. These results indicate that the transgene in the locus in chromosome 19 was not inherited through the generations of breeding in the line A4. Lane 1–3: DNA ladder, A4 PCR product, and WT PCR product, respectively.

**Additional file 4: Supplementary Figure 4.** Prenatal exposure to sodium arsenite or ethanol does not affect body, brain and liver weights. No differences were observed in the body weight (a), brain weight (b), and liver weight (c) between control, sodium arsenite-exposed, and ethanol-exposed groups at P20 [F(2,6) = 0.51, *P* = 0.62 (a), F(2,6) = 0.09, *P* = 0.92 (b), and F(2,6) = 0.24, *P* = 0.79 (c), all by one-way ANOVA].

## Data Availability

Whole-genome sequence data is available in SRA (SAMN16895777). The reporter transgenic mice will be made available from The Jackson Laboratory.

## Abbreviations

AP: Action potential
DIC: Differential interference contrast
EGFP: Enhanced green fluorescent protein
ER: Endoplasmic reticulum
EtOH: Ethanol
FASD: Fetal alcohol spectrum disorders
FLPo: Codon-optimized flippase recombinase
FRT: Flippase recombinase-recombination target
HS: Heat shock
HSE: Heat shock response element
i.p.: Intraperitoneal
RFP: Red fluorescent protein
sEPSC: Spontaneous excitatory post-synaptic current
WT: Wild-type
