# Supplementary figures and images for: Long-term spatial tracking of cells affected by environmental insults
Source: J Neurodev Disord. 2020 Dec 16;12:38. doi: 10.1186/s11689-020-09339-w (PMC7745478; doi:10.1186/s11689-020-09339-w)

# Supplementary Figure 1

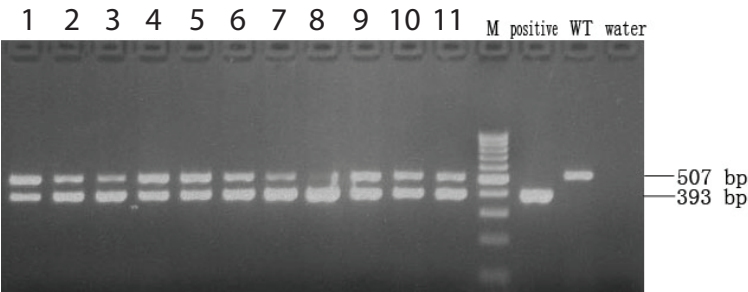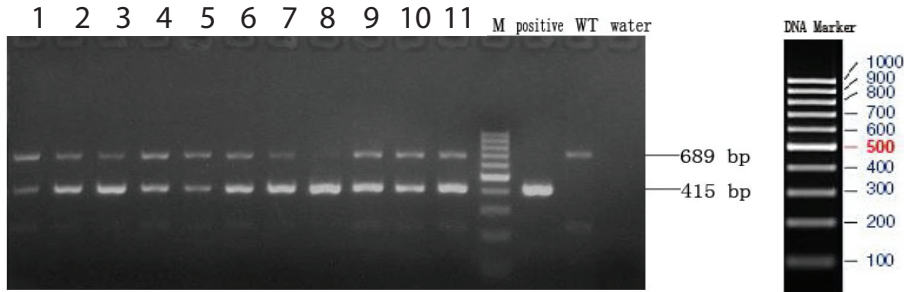

Supplement: Supplementary file 1 — Additional file 1: Supplementary Figure 1. Confirmation of transgene insertion in the founder lines. PCR was performed with two different primer sets using the tail tissue of founder lines (numbers 1–11 for lines A1-A11, respectively) for detection of the transgene (393 bp in upper panel, 415 bp in lower panel). The Rgs7 locus was used as the internal control (507 bp in upper panel, 689 bp in lower panel). [file 11689_2020_9339_MOESM1_ESM.pdf]

**a**

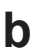

Supplement: Supplementary file 2 — Additional file 2: Supplementary Figure 2. Genomic locus of the transgene integration in chromosome 18 in the founder line A4. (a) Whole genome sequencing with 3X coverage defined an intronic region of Tcerg1 gene locus in chromosome 18 as the region of the transgene insertion in the F2 generation. The direction of the transgene in intronic regions is opposite to the transcriptional direction of the endogenous gene. Each one of the paired reads includes a partial sequence of FLPo. (b) PCR using the primer pair of FLPoF/chr18R after several generations of breeding (after the F5 generation) resulted in a ~ 1500 bp product (arrow) from the sample of founder line A4, confirming the transgene insertion in the identified locus. No PCR product was amplified from the sample of wild-type (WT) mice. Lane 1–4: DNA ladder, A4 genomic DNA input, A4 PCR product, and WT PCR product, respectively. [file 11689_2020_9339_MOESM2_ESM.pdf]

# Supplementary Figure 3

a

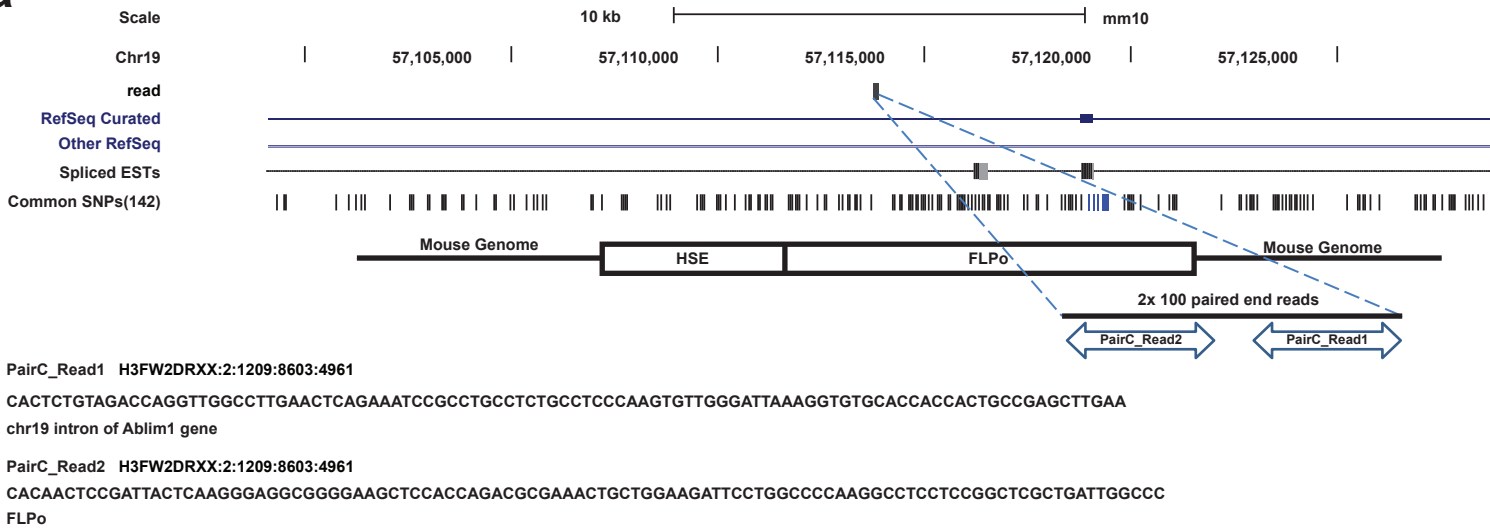

b

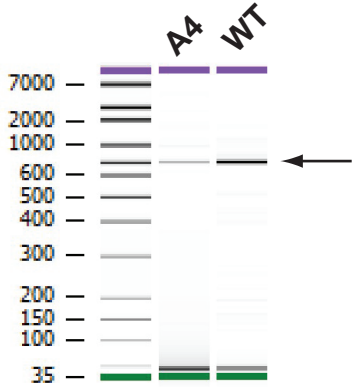

Supplement: Supplementary file 3 — Additional file 3: Supplementary Figure 3. Genomic locus of the transgene integration in chromosome 19 in the founder line A4. (a) Whole genome sequencing identified transgene insertion also in the 29 intronic region of Ablim1 gene in chromosome 19 in the F2 generation of A4. The direction of the transgene is opposite to the transcriptional direction of the endogenous gene. (b) PCR was performed to validate the insertion on the transgene in the founder line A4 using the primer pair of chr19F/chr19R after several generations of breeding (after the F5 generation). The size of PCR product detected by the BioAnalyzer was ~ 600 bp (arrow), which is expected to be amplified from the intrinsic genomic locus, as shown by the same size of product also amplified from the WT sample. Consistently, the product of > 2000 bp, which is expected to be amplified if the transgene is inserted, was not detected. These results indicate that the transgene in the locus in chromosome 19 was not inherited through the generations of breeding in the line A4. Lane 1–3: DNA ladder, A4 PCR product, and WT PCR product, respectively. [file 11689_2020_9339_MOESM3_ESM.pdf]

# Supplementary Figure 4

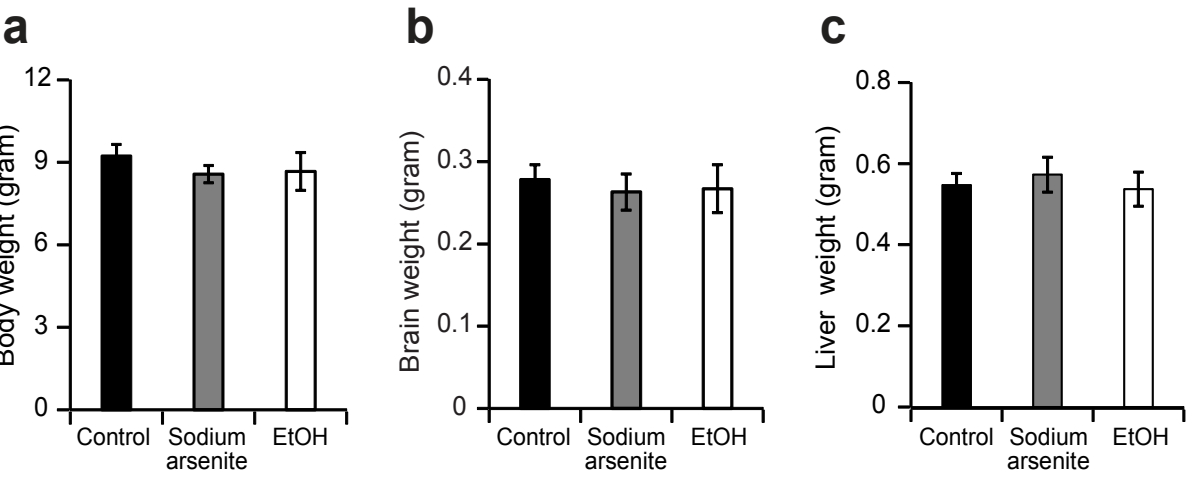

Supplement: Supplementary file 4 — Additional file 4: Supplementary Figure 4. Prenatal exposure to sodium arsenite or ethanol does not affect body, brain and liver weights. No differences were observed in the body weight (a), brain weight (b), and liver weight (c) between control, sodium arsenite-exposed, and ethanol-exposed groups at P20 [F(2,6) = 0.51, P = 0.62 (a), F(2,6) = 0.09, P = 0.92 (b), and F(2,6) = 0.24, P = 0.79 (c), all by one-way ANOVA]. [file 11689_2020_9339_MOESM4_ESM.pdf]
